# Frequency and Clinical Implication of the R450H Mutation in the Thyrotropin Receptor Gene in the Japanese Population Detected by Smart Amplification Process 2

**DOI:** 10.1155/2014/964635

**Published:** 2014-05-05

**Authors:** Katsuhiko Tsunekawa, Yoshimaro Yanagawa, Tomoyuki Aoki, Tadashi Morimura, Osamu Araki, Takao Kimura, Takayuki Ogiwara, Nobuo Kotajima, Masumi Yanagawa, Masami Murakami

**Affiliations:** ^1^Department of Clinical Laboratory Medicine, Gunma University Graduate School of Medicine, Maebashi 371-8511, Japan; ^2^Department of Health and Physical Education, Faculty of Education, Gunma University, Maebashi 371-8510, Japan

## Abstract

In Japanese pediatric patients with thyrotropin (TSH) resistance, the R450H mutation in TSH receptor gene (*TSHR*) is occasionally observed. We studied the frequency and clinical implication of the R450H mutation in *TSHR* in the general population of Japanese adults using smart amplification process 2 (SmartAmp2). We designed SmartAmp2 primer sets to detect this mutation using a drop of whole blood. We analyzed thyroid function, antithyroid antibodies, and this mutation in 429 Japanese participants who had not been found to have thyroid disease. Two cases without antithyroid antibodies were heterozygous for the R450H mutation in *TSHR*. Thus, the prevalence of this mutation was 0.47% in the general population and 0.63% among those without antithyroid antibodies. Their serum TSH concentrations were higher than the average TSH concentration not only in subjects without antithyroid antibodies but also in those with antithyroid antibodies. The R450H mutation in *TSHR* is relatively common in the Japanese population and potentially affects thyroid function. The present study demonstrates that the SmartAmp2 method is useful to detect the R450H mutation in *TSHR*, which is one of the common causes of TSH resistance in the Japanese population.

## 1. Introduction

The thyrotropin (TSH) receptor is a G-protein-coupled receptor that mediates TSH signaling during thyroid development, growth, and hormone synthesis. Loss-of-function mutations in the TSH receptor gene (*TSHR*) were first reported in patients with TSH resistance in 1995 [1]. Until now, various missense or nonsense mutations in* TSHR* have been identified in patients with TSH resistance [2]. Those patients were found to have euthyroid hyperthyrotropinemia or hypothyroidism, with a pattern of transmission consistent with autosomal recessive inheritance.

We previously reported the first cases of Japanese pediatric siblings with TSH resistance who were compound heterozygous for the R450H and G498S mutations in* TSHR* [3]. Subsequently, we reported three Japanese families with TSH resistance who were compound heterozygous for R450H and V473I, R450H and R519C, and R450H and R519G in* TSHR* [4]. To date, other pediatric patients with TSH resistance have been reported in Japan [5–7], and all Japanese patients with TSH resistance contained the R450H mutation in at least one allele, with the exception of one patient who was heterozygous for A204V mutation. The R450H mutation comprises substitution of Arg (CGC) with His (CAC) at codon 450, located in the first transmembrane domain of the TSH receptor. Transfection studies demonstrated that the R450H mutation resulted in moderately reduced TSH binding activity, moderately decreased cyclic adenosine monophosphate (cAMP) responses to TSH, and moderately decreased cell surface expression of TSHR. Although the R450H mutation in* TSHR* is occasionally observed in Japanese pediatric patients with TSH resistance, the frequency of this mutation in the general population of Japanese adults has not been reported until now.

Smart amplification process 2 (SmartAmp2) is a unique genotyping technology that can accurately detect mutations by a simple operation and within 30 minutes (min) under isothermal conditions using a drop of whole blood [8]. SmartAmp2 allows the analysis of mutations in many subjects, even newborns, from whom it is difficult to obtain a sufficient quantity of blood for genetic analysis.

In this study, we developed novel SmartAmp2 primer sets to detect the R450H mutation in* TSHR*, investigated the frequency of the R450H mutation in* TSHR*, and analyzed the association between this mutation and thyroid function in the general population of Japanese adults.

## 2. Materials and Methods

### 2.1. Subjects

Participants were recruited from an ongoing health survey for the evaluation of metabolic syndrome and glucose tolerance in six areas in Gunma Prefecture, Japan: Maebashi City, Shibukawa City, Ogo Town, Gunma Town, Nakanojo Town, and Yoshioka Town [9, 10]. We studied 429 participants (156 men, aged 26–89 years; 273 women, aged 22–83 years) without a significant goiter who had not been found to have thyroid disease based on interviews concerning lifestyle habits and medical history. Written informed consent was obtained from all the participants. This study was conducted with the approval of the Ethics Committee of Gunma University Graduate School of Medicine.

### 2.2. Physical and Blood Chemistry Examinations

Body weight was measured using the MC-190 Inner Scan (Tanita, Tokyo, Japan), and body mass index (BMI) was calculated as weight/height^2^ (kg/m^2^).

Venous blood samples were drawn from the participants after overnight fasting. Serum free 3,5,3′-triiodothyronine (FT_3_), free thyroxine (FT_4_), and TSH concentrations were analyzed by a chemiluminescent microparticle immunoassay on an Abbott ARCHITECT i2000SR Immunoassay Analyzer (Abbott Laboratories, Abbott Park, IL, USA). Serum antithyroglobulin antibody (TgAb) and antithyroid peroxidase antibody (TPOAb) were analyzed by an electrochemiluminescence immunoassay using Modular Analytics E170 (Roche Diagnostics, Indianapolis, IN, USA). Subjects with TgAb and TPOAb levels >28 IU/mL and >16 IU/mL, respectively, were considered to be positive cases.

### 2.3. Genotyping by the SmartAmp2 Method

The R450H mutation of* TSHR* was genotyped by the SmartAmp2 method [8, 10]. To test SmartAmp2 primers and evaluate the accuracy of genotyping, we used the plasmid templates of pSVL-*TSHR* wild type (wt) and pSVL-*TSHR* R450H, generated as described previously [3]. The whole blood samples, collected with the tube containing EDTA-3 K, and the genomic DNA samples, extracted from the whole blood by FlexiGene DNA Kit (Qiagen K.K., Tokyo, Japan), were used as the templates of the clinical samples. Whole blood samples were diluted threefold with 50 mM NaOH and denatured at 98°C for 3 min. The dried blood spot samples, punched to 3 mm diameter from the newborn screening card, or the buccal mucosa samples, which cut off the cotton swabs, were soaked in 20 *μ*L of 25 mM NaOH and denatured at 98°C for 3 min. The plasmid DNA and genomic DNA samples were directly denatured at 98°C for 3 min. After chilling on ice, the sample preparation (0.8 *μ*L of whole blood, dried blood spot, and buccal swab, 6,000 copies of plasmid DNA, or 8 ng of genomic DNA) was directly added into the reaction mixture (total volume of 10 *μ*L) containing 2.0 *μ*M folding primer (FP), 2.0 *μ*M turn-back primer (TP), 1.0 *μ*M boost primer (BP), 0.25 *μ*M of each outer primer (OP1 and OP2), 1.4 mM dNTPs, 20 mM Tris-HCl (pH 8.0), 10 mM KCl, 10 mM (NH_4_)_2_SO_4_, 8 mM MgSO_4_, 0.1% Tween20, 1 : 100,000 SYBR Green I (Takara Bio Inc., Otsu, Japan), and 2.4 units of* Aac* polymerase (K.K. DNAFORM, Yokohama, Japan). SmartAmp2 reaction mixtures were incubated at 60°C for 30 min under isothermal conditions using the 7500 Real-Time PCR Systems (Applied Biosystems, Forester City, CA, USA), where changes in the fluorescence intensity of SYBR Green I dye indicative of DNA amplification were monitored during the reaction. We detected the genotypes on the basis of the presence or absence of DNA amplification within 30 min.

**Figure 1:**
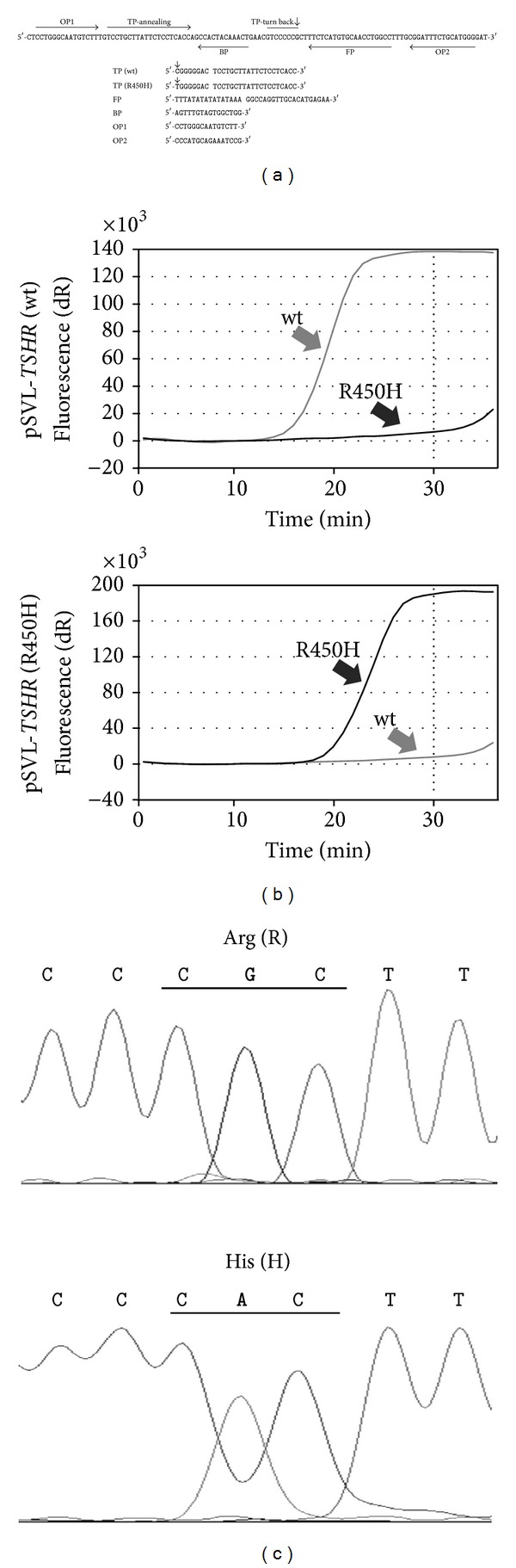
The primer annealing site design and amplification protocol for detecting the R450H mutation in* TSHR* by the SmartAmp2 method are shown. (a) Sequences and annealing sites for the SmartAmp2 primer set to detect the R450H mutation in* TSHR* are shown. An arrow indicates the nucleotide position of the R450H mutation in* TSHR*. TP: turn-back primer; FP: folding primer; BP: boost primer; and OP: outer primer. (b) Amplification curves generated with the SmartAmp2 primer set to detect the R450H mutation in* TSHR* using plasmid templates encoding pSVL-*TSHR* (wt) and pSVL-*TSHR* (R450H) are shown. (c) Direct sequencing results of the plasmid DNA templates pSVL-*TSHR* (wt) and pSVL-*TSHR* (R450H) are given.

To detect the R450H mutation in* TSHR*, we designed five primers termed TP, FP, BP, OP1, and OP2 for the SmartAmp2 method. We designed TPs to discriminate between 450R (CGC) and 450H (CAC) forms of* TSHR* and used TP (wt), FP, BP, OP1, and OP2 for the wt primer set and TP (R450H), FP, BP, OP1, and OP2 for the R450H primer set (Figure 1(a)).

We used the plasmid templates of pSVL-*TSHR* (wt) and pSVL-*TSHR* (R450H) to evaluate the accuracy of genotyping with the SmartAmp2 primer sets and then used verified primer sets to obtain amplification data for each plasmid (Figure 1(b)). We could successfully detect the R450H mutation on the basis of the presence or absence of DNA amplification within 30 min using each SmartAmp2 primer. Accurate detection by the SmartAmp2 method was confirmed by comparison with direct sequencing data (Figure 1(c)). Similar amplifications by the SmartAmp2 method were observed using the whole blood, the dried blood spot, and the buccal swab samples as templates without purification of the genomic DNA (data not shown).

### 2.4. Direct Sequencing

DNA sequencing of* TSHR* was performed as described previously [3]. Exons 1–10 of* TSHR* were amplified by PCR using plasmid DNA or genomic DNA samples as templates. Direct sequencing was performed using BigDye Terminator 3.1 (Applied Biosystems) after ExoSAP-IT treatment (GE Healthcare, Tokyo, Japan) and analyzed with a 3130xl Genetic Analyzer (Applied Biosystems).

### 2.5. Statistical Analysis

The results of each measurement are expressed as the mean ± standard deviation (SD). Binary logistic regression analysis was used to compare gender to the positive rate of antithyroid antibodies. Statistically significant differences between groups were tested using an unpaired Student's* t*-test or the Mann-Whitney *U* test as appropriate. All statistical analyses were performed using SPSS Statistics, version 20.0 (SPSS, Chicago, IL, USA).

## 3. Results

### 3.1. Thyroid Function and Antithyroid Antibodies in Participants

The physical and laboratory characteristics of 429 participants without a significant goiter who had not been found to have thyroid disease were shown in Table 1. Among the participants, there were 84 positive cases (19.6%) of TgAb, 63 positive cases (14.7%) of TPOAb, and 112 positive cases (26.1%) of antithyroid antibodies who had TgAb and/or TPOAb. The positive rate of each antibody was slightly but not significantly higher in women than in men (women (W) versus men (M): TgAb, 21.6% versus 16.0%; TPOAb, 15.0% versus 14.1%; and antithyroid antibodies, 28.6% versus 21.8%).

A comparison of thyroid function test for the negative and positive cases of antithyroid antibodies is shown in Table 2. Cases positive for antithyroid antibodies showed significantly higher serum TSH concentrations than the negative cases (*P* < 0.001). Seven cases positive for antithyroid antibodies showed elevated serum TSH concentrations above reference range. Of these, one case showed hypothyroidism, whose serum TSH concentration was 40.28 *μ*U/mL and serum FT_4_ concentration was 0.44 ng/dL, and six cases showed subclinical hypothyroidism, whose serum TSH concentrations were within 10 *μ*U/mL and serum FT_4_ concentrations were within reference range. On the other hand, all serum TSH concentrations for negative cases of antithyroid antibodies were within reference range. Serum FT_3_ and FT_4_ concentrations were not significantly different between the groups. Although these subjects had not been previously found to have thyroid disease, antithyroid antibodies were observed at a high frequency and were associated with hyperthyrotropinemia in the present study.

### 3.2. Frequency of the R450H Mutation in* TSHR* and Its Effect on Thyroid Function

**Figure 2:**
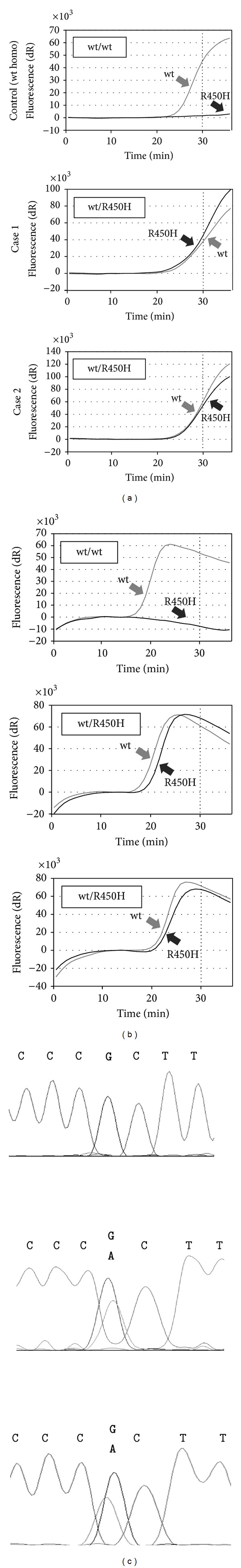
Detection of the R450H mutation in* TSHR* by the SmartAmp2 method using the clinical samples. (a) Amplification curves generated using whole blood from the participants by the SmartAmp2 method are shown. (b) Amplification curves generated using genomic DNA from the participants by the SmartAmp2 method are shown. (c) Direct sequencing results generated using genomic DNA of the participants are shown.

We analyzed the R450H mutation in* TSHR* by the SmartAmp2 method using whole blood and genomic DNA samples. Among the 429 samples, DNA amplification by both the wt and R450H primer sets was observed in two samples (Figures 2(a) and 2(b)). It was demonstrated that these two cases were heterozygous for wt/R450H in* TSHR*. Direct sequencing also confirmed heterozygosity for wt/R450H in two cases (Figure 2(c)). Two cases did not have TgAb and TPOAb. The prevalence of the R450H mutation was 0.47% among the whole participants and 0.63% among the participants without antithyroid antibodies.

**Figure 3:**
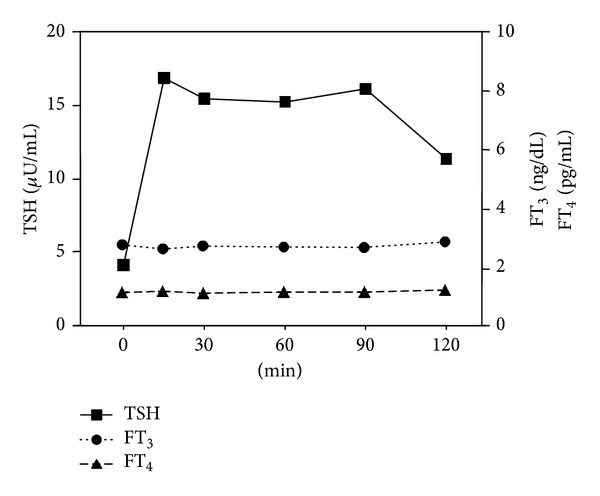
TRH provocative test results from a carrier of the R450H mutation in* TSHR*.

The clinical characteristics and thyroid function tests for these two cases are shown in Table 3. Case 1 was a 77-year-old man who received glimepiride and metformin to treat type 2 diabetes. Case 2 was a 71-year-old man who received valsartan and benidipine to treat hypertension. Neither case had a goiter. Their serum FT_3_ and FT_4_ concentrations were within the reference range. In case 1, the serum TSH concentration was 2.61 *μ*U/mL. In case 2, repetitive measurements of the serum TSH concentration yielded values of 3.68, 6.87, 4.13, and 5.37 *μ*U/mL, which occasionally exceeded the reference range. Although neither had detectable TgAb or TPOAb, their serum TSH concentrations were higher than the average TSH concentration not only in subjects without antithyroid antibodies but also in those with antithyroid antibodies. In the TSH-releasing hormone (TRH) provocative test of case 2, prolonged TSH response was observed [11], although the serum FT_3_ and FT_4_ concentrations did not increase (Figure 3). The TRH provocative test could not be performed on case 1.

## 4. Discussion

In the present study, we developed novel SmartAmp2 primer set to detect the R450H mutation in* TSHR* from a drop of whole blood. Using the primer set, we analyzed the frequency of the R450H mutation in the general population of Japanese adults and the effect of the mutation on the thyroid function in the carriers of this mutation. This appears to be the first report to investigate the R450H mutation in a Japanese adult cohort that was not found to have thyroid disease.

To date, five studies have demonstrated the Japanese pediatric patients with TSH resistance, who have carried the R450H mutation in at least one allele except one patient. Furthermore, the R450H mutation in* TSHR* was also detected in patients with TSH resistance in various countries in East Asia, including China [12], Korea [13], and Taiwan [14], although this mutation has not been reported in Caucasian populations. In the Welsh population, two heterozygous W546X mutations were detected in 368 individuals, and the W546X mutation was suggested to be a potential major contributor to hypothyroidism [15]. In the present study, we similarly detected two heterozygous R450H mutations in 429 Japanese adult individuals. In addition, we observed the antithyroid antibodies (TgAb and/or TPOAb) in 112 subjects (26.1%). The increased prevalence of antithyroid antibodies was reported in elderly subjects [16]. The high frequency of patients positive for antithyroid antibodies may be, at least in part, due to the inclusion of many elderly participants (aged 64.4 ± 10.1 years) in this study. The prevalence of R450H mutation was 0.47% among the whole participants and 0.63% among the participants without antithyroid antibodies.

The association between the R450H mutation in* TSHR* and thyroid function has not been reported in adult patients. We previously reported that the R450H mutation led the receptor function to the moderate impairment of cAMP response to TSH, TSH binding activity, and cell surface expression [4]. In this report, the patients with TSH resistance were all compound heterozygous for R450H and either V473I, R519G, or R519C. The impairment of receptor function in vitro (R519G > R519C > R450H > V473I) related closely to serum TSH levels in patients bearing the following genotypes: R519G/R450H > R519C/R450H > V473I/R450H. In the present study, sequencing analysis of exons 1–10 in* TSHR* revealed that both cases were heterozygous for wt/D727E, a polymorphism with little effect on thyroid function [17]. Despite the absence of antithyroid antibodies in two R450H mutation carriers, the serum TSH concentrations of case 2 occasionally exceeded reference range and were higher than the average TSH concentrations in subjects with antithyroid antibodies. Even in case 1, his serum TSH concentration was higher than the average TSH concentration not only in subjects without antithyroid antibodies but also in those with antithyroid antibodies. These results suggest that heterozygosity for the R450H mutation potentially causes TSH resistance in Japanese adults.

Whereas the previous studies detected the mutations in* TSHR* by direct sequencing analysis [1–8], we used the SmartAmp2 method to detect the R450H mutation in* TSHR* for the first time. Using the SmartAmp2 method, all processes can be performed by a simple operation because the initial purification of genomic DNA from blood is not necessary and mutations can be directly detected on the basis of the presence or absence of DNA amplification. Additionally, accurate amplification is achieved by reduction of nonspecific amplification using a unique asymmetrical primer set. Because the SmartAmp2 method is a simple and reliable method to detect mutations, it is often utilized in clinical settings, for example, for highly sensitive detection of the* EGFR* mutation in lung cancer [18], rapid and highly sensitive detection of the 2009 pandemic influenza A (H1N1) virus [19], and simple detection of multiple polymorphisms of the **β*3AR*, **β*2AR*, and* UCP1* genes in many subjects [10]. Notably, the SmartAmp2 method enables us to analyze mutations using a drop of whole blood and the dried blood spot. Therefore, this method is useful to detect mutations in newborns, from whom it is difficult to obtain a sufficient quantity of blood. We believe that the SmartAmp2 method to detect the R450H mutation in* TSHR* will be a valuable tool for detailed examination of the cause of hyperthyrotropinemia after newborn screening in Japan. Furthermore, this method will also be useful to investigate the cause of hyperthyrotropinemia or hypothyroidism in East Asia.

We studied the frequency of the R450H mutation of* TSHR* in Japanese general population. A limitation of the present study was the presence of only two subjects with the R450H mutation, which was not enough to investigate the clinical implication of the R450H mutation. It will be necessary to analyze the frequency and the clinical implication of the R450H mutation in Japanese subjects with elevated TSH. Furthermore, it will be of interest to elucidate whether this mutation is observed beyond East Asian populations.

## 5. Conclusions

In conclusion, we identified two cases of heterozygous R450H mutations in* TSHR* among 429 Japanese adults using the SmartAmp2 method. The prevalence of the R450H mutation was 0.47% in the general population and 0.63% among those without antithyroid antibodies. One of the R450H carriers exhibited mild hyperthyrotropinemia and tended to have TSH resistance. These results suggest that the R450H mutation in* TSHR* is relatively common and potentially affects thyroid function in Japanese carriers. The present study demonstrates that the SmartAmp2 method to detect the R450H mutation in* TSHR* is a useful tool for examining the cause of hyperthyrotropinemia in Japanese and East Asian populations.

## Figures and Tables

**Table 1:** Physical and laboratory characteristics of the participants.

	Men	Women	Total	Reference range	*P*
Number	156	273	429		
Age (years)	66.4 ± 10.8	63.2 ± 9.5	64.4 ± 10.1		<0.001
BMI (kg/m^2^)	23.4 ± 2.5	22.6 ± 2.9	22.9 ± 2.8		<0.001
TSH (*μ*U/mL)	1.60 ± 0.97	2.10 ± 2.63	1.92 ± 2.19	0.35–4.94	0.001
FT_3_ (pg/mL)	3.10 ± 0.46	3.11 ± 0.51	3.11 ± 0.49	1.71–3.71	0.897
FT_4_ (ng/dL)	1.04 ± 0.16	1.06 ± 0.16	1.05 ± 0.16	0.70–1.48	0.185
Positive cases of TgAb	25 (16.0%)	59 (21.6%)	84 (19.6%)		0.162
Positive cases of TPOAb	22 (14.1%)	41 (15.0%)	63 (14.7%)		0.797
Positive cases of antithyroid antibodies	34 (21.8%)	78 (28.6%)	112 (26.1%)		0.125

FT_3_: free 3,5,3′-triiodothyronine.

FT_4_: free thyroxine.

TgAb: antithyroglobulin antibody.

TPOAb: antithyroid peroxidase antibody.

Positive cases of antithyroid antibodies had TgAb and/or TPOAb.

Data are expressed as mean ± standard deviation (SD).

*P* value was analyzed by comparing men with women.

**Table 2:** Thyroid function in negative or positive cases of antithyroid antibodies.

	Antithyroid antibodies		*P*
	Negative	Positive	
Number*	122/195	34/78	
Age (years)	63.7 ± 10.7	66.3 ± 7.8	0.063
BMI (kg/m^2^)	22.9 ± 2.8	23.0 ± 2.7	0.605
TSH (*μ*U/mL)	1.69 ± 0.93	2.58 ± 3.92	<0.001
Number of cases with elevated TSH levels above reference range	0	7	
FT_3_ (pg/mL)	3.12 ± 0.49	3.08 ± 0.48	0.419
FT_4_ (ng/dL)	1.05 ± 0.16	1.04 ± 0.17	0.349

*Men/women. Data are expressed as mean ± SD.

**Table 3:** Thyroid function and antithyroid antibodies in carriers of the R450H mutation in *TSHR*.

Test day	Case 1	Case 2				Reference range
	April 2008	March 2008	April 2009	May 2009	June 2010	
Age (years)	77	71				
Gender	Man	Man				
Disease	Type 2 DM	Hypertension				
BMI (kg/m^2^)	23.4	24.6				
TSH (*μ*U/mL)	2.61	3.68	6.87	4.13	5.37	0.35–4.94
FT_3_ (pg/mL)	3.10	3.00	2.40	2.51	2.57	1.71–3.71
FT_4_ (ng/dL)	1.08	1.22	0.96	1.11	1.10	0.70–1.48
TgAb (IU/mL)	11.3	17.9				<28
TPOAb (IU/mL)	<5.0	<5.0				<16

Type 2 DM: type 2 diabetes mellitus.
